# Using a novel hybrid AI-based paper-to-digital pipeline for extracting patient-level clinical data from routine hospital records in Kenya: a retrospective multicentre study

**DOI:** 10.1136/bmjdh-2026-000092

**Published:** 2026-05-22

**Authors:** Timothy Tuti, Franklin Okech, Niels Peek, Janette Karimi, Muthoni Ogolla, Cynthia Khazhenzi, Mike English

**Affiliations:** 1 KEMRI-Wellcome Trust Research Programme Nairobi, Nairobi, Nairobi County, Kenya; 2 The Healthcare Improvement Studies Institute, Department of Public Health and Primary Care, University of Cambridge, Cambridge, England, UK; 3 Division of Reproductive Maternal, Newborn Child and Adolescent Health (RMNCAH), Kenya Ministry of Health, Nairobi, Nairobi County, Kenya; 4 Centre for Tropical Medicine and Global Health, University of Oxford, Oxford, UK

**Keywords:** Artificial intelligence, Medical Records, User-Centered Design, Global Health, Access to Information

## Abstract

**Objective:**

This study explored how hybrid paper-to-digital approaches could reduce time and effort for data capture and reporting in settings with low electronic health record (EHR) adoption while increasing the volume and diversity of routine patient-level clinical data within health information systems.

**Methods and analysis:**

We used human-centred design to co-design two components with nurses, doctors and records officers from newborn units in eight geographically dispersed Kenyan hospitals: (1) machine-readable versions of routinely used patient transfer, admission and discharge forms (February–October 2024) and (2) a minimum viable paper-to-digital clinical data pipeline (November 2024–July 2025). Participating hospitals piloted the machine-readable paper forms in routine clinical settings between November 2024 and May 2025 with all newborn unit admissions eligible for inclusion. We used artificial intelligence (AI) models within the co-designed pipeline to auto-extract clinical data from forms, with models being continuously evaluated and updated.

**Results:**

Of the 7118 patients admitted to newborn units across participating hospitals, 6482/7118 (91.1%) had at least one designed form in their patient files, with 1615 clinicians of different cadres having used the forms. The clinical data pipeline extracted checkbox fields with aggregate scores of 99.25% (95% CI 98.98% to 99.45%) for the area under the receiver operating characteristic curve (AUCROC), 98.91% (95% CI 98.49% to 99.21%) for F1-score, 98.85% (95% CI 98.32% to 99.22%) for positive predictive value and 99.02% (95% CI 98.62% to 99.30%) for sensitivity. For the free-text fields, the aggregate scores for exact accuracy, character error rate and word error rate across all hospitals were 95.66% (95% CI 94.24 to 96.74%), 1.66% (95% CI 1.21% to 2.26%) and 4.34% (95% CI 3.26% to 5.76%), respectively. The hybrid approach reduced human time required for manual data extraction by 50% and forms requiring data correction by 60%.

**Conclusion:**

Our co-designed novel paper-to-digital pipeline with AI-based extraction from routine paper records achieved high accuracy and substantial efficiency gains, offering a scalable solution for generating patient-level data in routine clinical settings in Sub-Saharan Africa where EHRs remain out of reach or are difficult to implement.

**WHAT IS ALREADY KNOWN ON THIS TOPIC**

- Limited electronic health records (EHR) adoption across Sub-Saharan Africa (SSA) leaves public hospitals reliant on poor aggregate summaries and scant patient-level data to enable risk stratification, outbreak detection, care quality assessment and treatment outcome monitoring.

**WHAT THIS STUDY ADDS**

- The hybrid artificial intelligence (AI)-enabled paper-digital pipeline improved efficiency, reducing manual extraction time by ~36% and data correction needs by ~60%, while achieving high accuracy across checkbox and free-text data fields.
- External hospital validation showed strong generalisability across heterogeneous neonatal units, establishing a validated minimum viable hybrid paper-to-digital design for deployment in similar low-resource hospital settings.

**HOW THIS STUDY MIGHT AFFECT RESEARCH, PRACTICE OR POLICY**

- Accurate patient-level data from non-EHR settings using AI-enabled paper-digital pipeline can enable fairer benchmarking of hospital performance across countries and regions in SSA after adjusting for case mix.
- The pipeline enables scalable extraction of previously difficult-to-get longitudinal paper-based clinical data (eg, treatment data from treatment sheets), supporting large-scale analyses in areas such as antimicrobial stewardship and resistance.

## Introduction

Routine health information systems (RHISs) are an essential building block of learning health systems (LHS), where to facilitate organisational learning, data must be generated from routine clinical practice, knowledge must be generated from that data and the knowledge must be fed back into practice.^1^ Institutionalising use of routine data from RHIS is essential for consistent decision-making on (1) the quality of hospital care, (2) allocation of resources in health and (3) quality improvement and benchmarking activities, etc, and ensures decision processes are sustainable, durable and adapt well to dealing with emergent issues in the health systems such as pandemics and/or effects of climate change on health outcomes.^2–4^ However, the use of RHIS for routine system performance monitoring is needed in Sub-Saharan Africa (SSA), as highlighted especially during the COVID-19 pandemic,^5^ although some donor-dependent resource-intensive ‘vertical’ sub-systems show better performance (eg, for HIV, TB).^6^ The poor clinical data pipelines from SSA propagate an over-reliance on complex model-based estimates of disease burden that are fraught with inconsistencies and bring significant uncertainty into decision-making.^7 8^

The lack of quality clinical data pipelines is especially critical in SSA. For example, Maternal, Perinatal and Neonatal Death Surveillance and Response (MPNDSR) is a global Integrated Disease Surveillance and Response (IDSR) priority.^9–13^ SSA is estimated to account for 43% of the 2.4 million global neonatal deaths annually, with preterm births/low birth weight, birth defects and increasingly antimicrobial resistance being likely leading causes.^14–16^ However, accurate national data to track progress are typically lacking.

### The current need for an alternative to comprehensive electronic medical record systems

Electronic health records (EHRs) are the ideal long-term solution for leveraging patient-level data from routine hospital care for MPNDSR/IDSR and other decision-making activities. However, their full implementation in most SSA countries is unlikely at scale in the short-term and even medium-term with implementation challenges such as:

1. The cost of initial implementation of EHRs per provider/hospital is high and even higher when the maintenance cost is factored in, making them inaccessible at scale in resource-poor settings.^17–20^
2. The infrastructure (electricity, internet, computers, etc), human resource and data security capacity needed to successfully implement and run EHRs are required in many urban and rural regions in SSA.^18 20 21^
3. Inpatient use-cases that include multiple stages in care over many days are the most difficult to implement and least developed components of EHRs.^22 23^

However, there is an urgent need for high-quality individual patient ‘row-level’ data (eg, demographics, clinical danger signs, diagnoses and treatments provided, etc) from hospital care from SSA, including Kenya. This would enable mortality risk stratification by demographic characteristics and diagnoses, help identify disease outbreaks and clusters of emergent diseases or new patient ‘phenotypes’ and enable examination of care quality (eg, intervention coverage) or treatment outcomes (eg, relevant to antimicrobial resistance). Currently in Kenya, in the absence of EHR adoption at scale, only aggregate indicator data are collated at scale from hospitals to inform routine decision-making (eg, the District Health Information System V.2 (DHISv2)^24^).

DHISv2 is the most common reporting platform in SSA functioning as a large-scale surveillance tool.^24^ To populate DHISv2, health records information officers (HRIOs) and healthcare workers (HCWs) must physically review patient paper records and registers, code and aggregate data, then manually enter the summary statistics onto the reporting platform. This is time-consuming for personnel in over-stretched and under-resourced facilities, with these processes often also suffering from task duplication.^25^ The consequence has been poor DHISv2 data with limited evidence of meaningful use of hospital data (eg, decision-making activities),^18 24 26^ while the need for anonymised patient-level data at scale still persists. We need now an efficient, effective, context-appropriate clinical data-pipeline that works at scale even in remote rural areas in SSA that can integrate with DHISv2 and subsequently with emerging lower cost EHRs while modelling the requirements for clinically meaningful EHR implementation.

### Potential of hybrid paper-to-digital data capture systems

To address this challenge while fostering a culture of information acquisition and use at all levels of the health system, hybrid paper-digital approaches are a solution. These can make best use of existing resources and practices, such as structured paper facility registers or individual records and leverage artificial intelligence (AI) and machine learning (ML) approaches (eg, object detection deep learning models for information retrieval).^27 28^ These hybrid paper-digital approaches offer time-saving and effort-saving benefits to staff plus the ability to capture a much wider scope of patient-level data on every case while requiring significantly less resource (eg, internet access, computer terminals) and labour (eg, training) relative to EHR implementation.

### The need to improve data use

Even if hybrid paper-digital approaches exist at scale, they may not generate quality clinical data, because HCWs, hospitals and policy makers have a finite capacity to engage with, respond to and act on insights from data. To create valued information systems, we need to unearth how to optimise provision and utilisation of information that is valuable to all stakeholders whose needs vary across system levels. Within the hospital context, use of human-centred design approaches could be leveraged to develop tools and data that are of value to different stakeholders^29 30^ as an essential step to later leverage audit and feedback for improving reporting rates in DHISv2 or other systems.^24 26^ If mechanisms for data use for action are well targeted^31^ and the feedback recipients receive the information they need, this may create a virtuous cycle.^32^

## Objectives

The study objectives were to explore how hybrid approaches to designing and digitising paper records could be leveraged to reduce the time and effort required for data capture and reporting in RHIS. In particular:

- To co-design machine-readable clinical forms and data extraction processes that enable under-resourced health facility personnel to meet reporting requirements while producing high-quality patient-level data.
- To evaluate the accuracy and efficiency of AI-based data extraction from the co-designed forms compared with the current manual data abstraction.

Addressing the above challenges would support surveillance by reducing data missingness and unreliability due to poorly designed data collation and reporting systems, improving data scope by incorporating (more) individual patient-level data at scale and enhancing its value for action by advancing an information-use culture.^24 25 33–35^ This research’s goal was to unearth a minimum viable scalable approach to a nation-wide informatics pipeline embedded within existing hospital data platforms, capable of supporting a LHS approach.^1^

## Methodology

### Study design, setting and context

This implementation and usability research was embedded within the clinical information network (CIN), a LHS approach between the KEMRI-Wellcome Trust Research Programme (KWTRP), the Ministry of Health (MoH), Kenya Paediatric Association and 21 partner hospitals to facilitate better data use for decision making in neonatal care. The hospitals in CIN are first referral-level, geographically dispersed hospitals with an IQR of annual newborn unit (NBU) inpatient admissions between 550 and 1640. Indepth description of the development of CIN and its activities is detailed elsewhere.^36–38^ This study took advantage of Kenya’s MoH and other health technical partners’ efforts to institutionalise the use of a standardised newborn inpatient booklet across all hospitals providing inpatient newborn care across the country by co-opting the MoH timelines for the study’s milestone targets (online supplemental table 1). The contents of the booklet are discussed at length in the Data sources and management section.

10.1136/bmjdh-2026-000092.supp2 Supplementary data

### Study size and participants

Due to budgetary constraints, only 8/21 CIN hospitals were included in this study, selected to represent the facilities with the largest NBUs. All neonates aged ≤28 days admitted to the NBUs between November 2024 and May 2025 in the 8/21 CIN hospitals were eligible for inclusion.

### Data sources and management

Methods of data collection and cleaning in the CIN are reported in detail elsewhere.^38^ Clinical data for neonatal admissions were captured through Neonatal Admission Record (NAR) forms and other forms and charts that are part of the hospital’s medical record. The NAR and associated patient charts are filled by medical personnel and clinicians are prompted with a checklist of fields covering nine documentation domains that include demographics, admission information, maternal history, presenting complaints, cardinal signs on examination, other physical examinations, vital signs and proposed forms of supportive care.^39^ Other charts that were also used included a comprehensive newborn monitoring chart (collects data on vital signs, feeds and fluids prescribed) which was developed and introduced between March and June 2019,^30^ internal transfer forms (completed by nurses/midwives containing key data when a baby is transferred internally from maternity unit to NBU), treatment sheets, discharge summaries (typically completed by medical personnel) and death notification reports in case death occurs. The clinical signs included in the NAR are based on recommendations in guidelines from the national MoH and the WHO.^40^

NAR forms were originally developed as part of the Emergency Treatment and Triage plus admission training approach for essential inpatient newborn care.^30 39^ This study reviewed three paper forms in the neonatal inpatient booklet (the internal transfer, NAR and discharge summary forms) with their machine-readable versions^41^ iteratively co-designed with paediatricians, nurses and HRIOs from participating CIN hospitals between February and October 2024. Machine-readable paper forms are designed for ease of computerised processing by using specific design, layouts, fonts and optical fiducial marks that can be scanned and interpreted automatically.^41^ The machine-readable forms were provided to hospitals during the study, and subsequently, the hospital teams had the discretion of continuing to use them after the study support using hospitals’ own resources.

Each hospital had a HRIO who extracted data from the machine-readable forms (the internal transfer forms, the NAR and the discharge summary) into a Research Electronic Data Capture (REDCap) database immediately after patient discharge throughout the study period.^42^ Two sets of data were captured: de-identified patient-level datasets and de-identified clinician data capturing the unique scribe (ie, clinician) who used the paper forms. The patient-level dataset contained comprehensive data on admission details, patient history, clinical investigations, treatment and discharge information including diagnoses and outcome. The HRIOs also used low-cost scanners (~US$150) to scan and upload the machine-readable paper forms onto a secured KWTRP server (online supplemental figure 1). All abstracted data were subjected to comprehensive data quality assurance (DQA) checks through a custom web application where the data the HRIO entered into REDCap were overlayed on the scanned image for the HRIO to verify its accuracy (online supplemental figure 2).

10.1136/bmjdh-2026-000092.supp1 Supplementary data

### Quantitative variables

The outcomes of interest for the paper-to-digital data pipeline were (a) whether checkboxes were either selected or unselected (ie, binary) and the value of the character entered for prespecified fields of interest (ie, multiclass). The checkboxes and text of interest were grouped and analysed at field level. The text variables included vital signs, test results (eg, random blood sugar results) and patient demographics (eg, age, parity, weight, etc). Blank fields were treated as missing values.

### Statistical methods

Scanned images were processed according to the steps highlighted in online supplemental table 2. Image pre-processing was achieved using OpenCV library^43^ in Python programming language (V.3.11). The extracted regions of interest were fitted using the ResNET-50 which is a 175-layer deep convolutional neural network for image classification which had been pre-trained on the ImageNet dataset, a process known as transfer learning. Layers 26–175 of ResNET-50 were unfrozen and fine-tuned using the scanned image dataset from CIN coupled with the respective DQA verified REDCap dataset. To minimise overfitting and bias, early stopping was used for the number of epochs during model training, with 30% of the data being used as a hold-out dataset for internal validation and with 50% of the hidden layer units dropped in the final hidden layer before the model’s output layer,^44 45^ with the process explained further in online supplemental table 3.

To examine the heterogeneity in model performance, we compared the models' internal-external cross-validation (IECV) performance where we omitted one hospital at a time using it as the validation dataset, built the model on the remaining hospitals and evaluated the model’s performance on the hospital left out. We repeated this process with each iteration using a different hospital as the validation dataset.^46^ The performance measures resulting from IECV were pooled using random-effects meta-analysis,^46–49^ an approach that accounts for the precision of hospital-specific performance estimates while quantifying between-hospital variability (heterogeneity) of model performance, with heterogeneity quantified by the between-hospital SD of model performance (Tau, (τ)).^50^ Meta-analysis results were reported as point estimates with 95% CIs, indicative of the precision of the model’s average performance across all hospitals and accounts for heterogeneity between hospitals and therefore indicates what performance can be expected when the model is applied within a specific hospital.

The models’ performance was evaluated on the validation dataset with F1 score (the balance between precision and recall for a model), positive predictive value (of all the checkboxes that the model identified as being selected, how many were truly selected), sensitivity (of the truly selected checkboxes, how many did the model identify) and AUCROC (how well a model can tell the difference between selected and unselected checkboxes) being used as the evaluation metrics for checkbox fields. Exact accuracy, character error rate (CER) and word error rate (WER) metrics were used for text, after grouping by field. The CER and WER metrics were normalised to be between 0% and 100%, giving an indication of the minimum number of operations to transform the model output into the ground truth text, with lower values being better.^51^

### Sensitivity analyses

We conducted sensitivity analysis to evaluate how many records would have been necessary to ensure the models performed well. We varied the number of machine-readable records used in model training and using IECV evaluated model performance on the dataset from the remaining hospitals reporting the previously mentioned performance metrics.

### Interoperability analyses

As this study was designed to design and implement a hybrid-paper-to-digital pipeline, interoperability could only be achieved once data have been digitised. The results outlined here are the initial step to digitise data, with future work looking to develop a health data exchange framework applying Health Level Seven (HL7) Fast Healthcare Interoperability Resources (FHIR) technical standards to share data with the national reporting platform DHISv2 as illustrated by the ongoing efforts highlighted in online supplemental table 1.

### Patient and public involvement

It was not possible to involve patients or the public in this study’s design, conduct, reporting or dissemination plans. However, SERU consists of patient and public representatives to ensure they are involved in granting ethics approval.

## Results

### Descriptive findings

**Figure 1:**
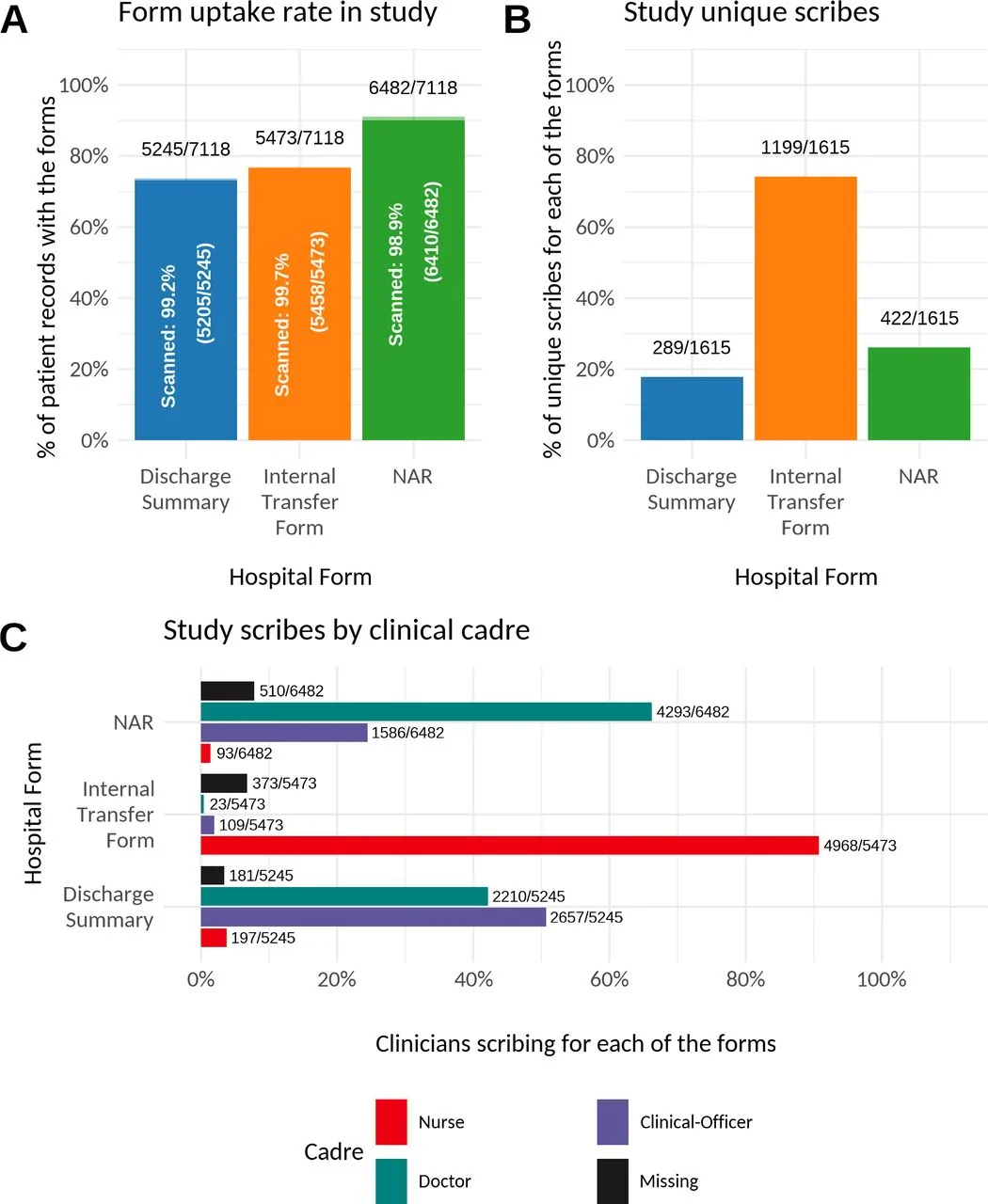
Machine-readable paper forms used in this study. NAR, Neonatal Admission Record.

A total of 7118 patients were included in the study across the participating hospitals with 6482/7118 (91.1%), 5473/7118 (76.9%) and 5245/7118 (73.7%) having the machine-readable NAR, internal transfer form and discharge summary used on them, respectively, during the inpatient stay (figure 1). A total of 17 200 machine-readable forms were used with 17 074 (99.27%) later scanned for the modelling tasks, though 5004/17 074 (29.31%) were rejected due to poor quality. Discharge summaries made up 4200/5004 (83.93%) of the rejected scans since they used carbon copies of paper records which had barely visible documentation, even by humans. A total of 1615 unique clinicians (scribes) of different cadres (doctors, nurses and clinical officers (ie, physician associates)) used the machine-readable forms while reviewing the patients (figure 1) with >70% of the demographic and signs/symptoms fields filled in these source documents (online supplemental figure 3). Interventions and vital signs fields had variable documentation levels in the source documents varying between 50% and 80% completed (online supplemental figure 3).

### Quality of data the models extract

**Figure 2:**
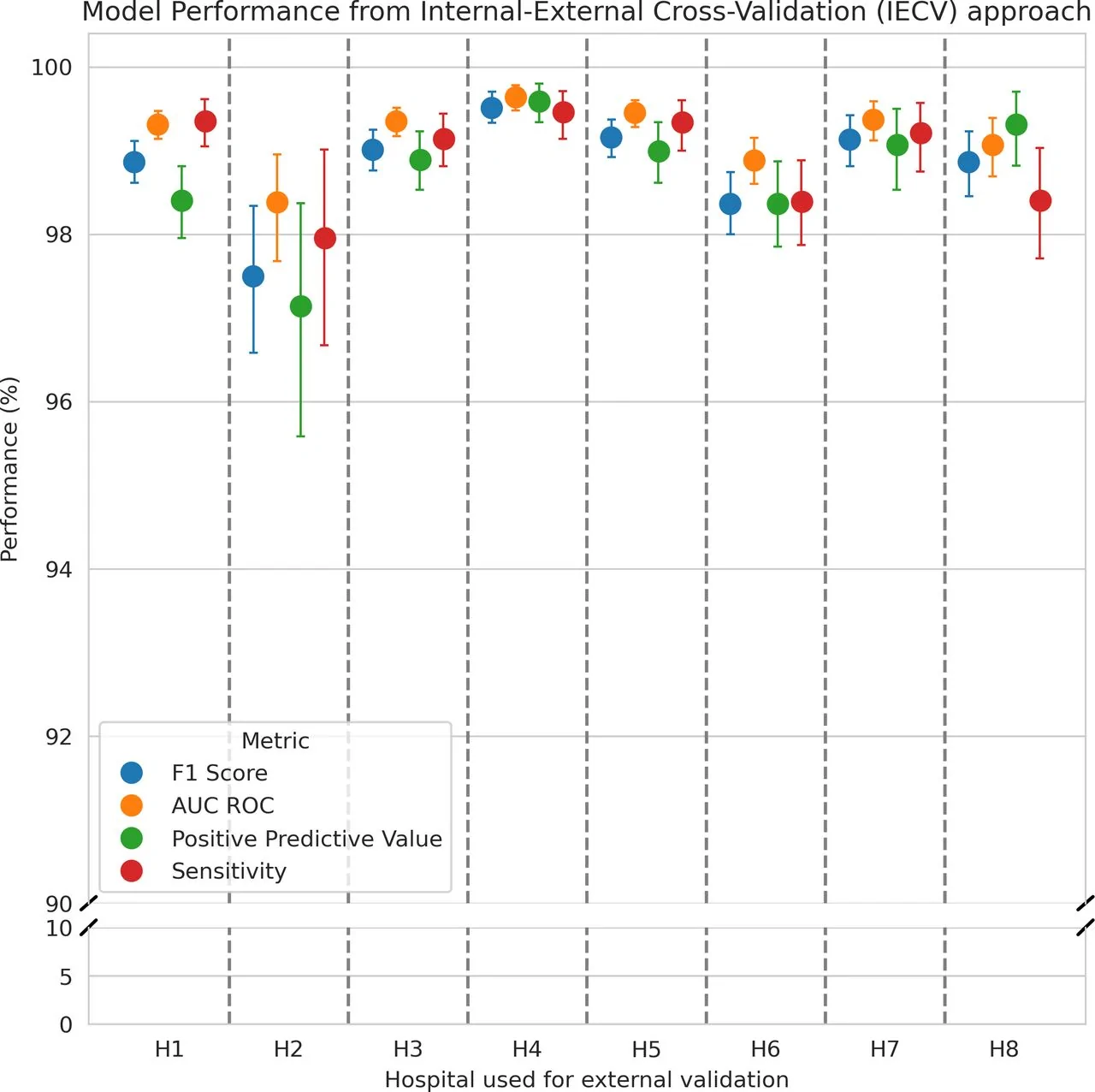
Model performance from IECV approach in predicting checkbox fields. AUROC, area under the receiver operating characteristic curve; IECV, internal-external cross-validation.

For all models, the summary estimates were obtained using random effects meta-analysis with the SE and between-study heterogeneity (τ) is given on the scale of the meta-analysis (eg, logits for AUCROC, F1 Score, positive predictive value and sensitivity, exact accuracy) (table 1). The accuracy of the models’ performance in extracting checkbox data was high across all metrics of interest and across all hospitals, with all having 95% CIs above 98% (table 1, figure 2).

**Table 1:** Meta-analysis results of model performance

Metric	Summary estimate	95% CI	SE	Tau
Checkbox fields				
AUCROC	99.25%	98.98% to 99.45%	0.1054	0.4100
F1 Score	98.91%	98.49% to 99.21%	0.1174	0.4406
Positive predictive value	98.85%	98.32% to 99.22%	0.1677	0.5089
Sensitivity	99.02%	98.62% to 99.30%	0.1303	0.4323
Free-text fields				
Exact accuracy	95.66%	94.24% to 96.74%	0.0979	0.3976
Character error rate	1.66%	1.21% to 2.26%	0.1107	0.4178
Word error rate	4.34%	3.26% to 5.76%	0.0979	0.3976

AUCROC, area under the receiver operating characteristic curve.

**Figure 3:**
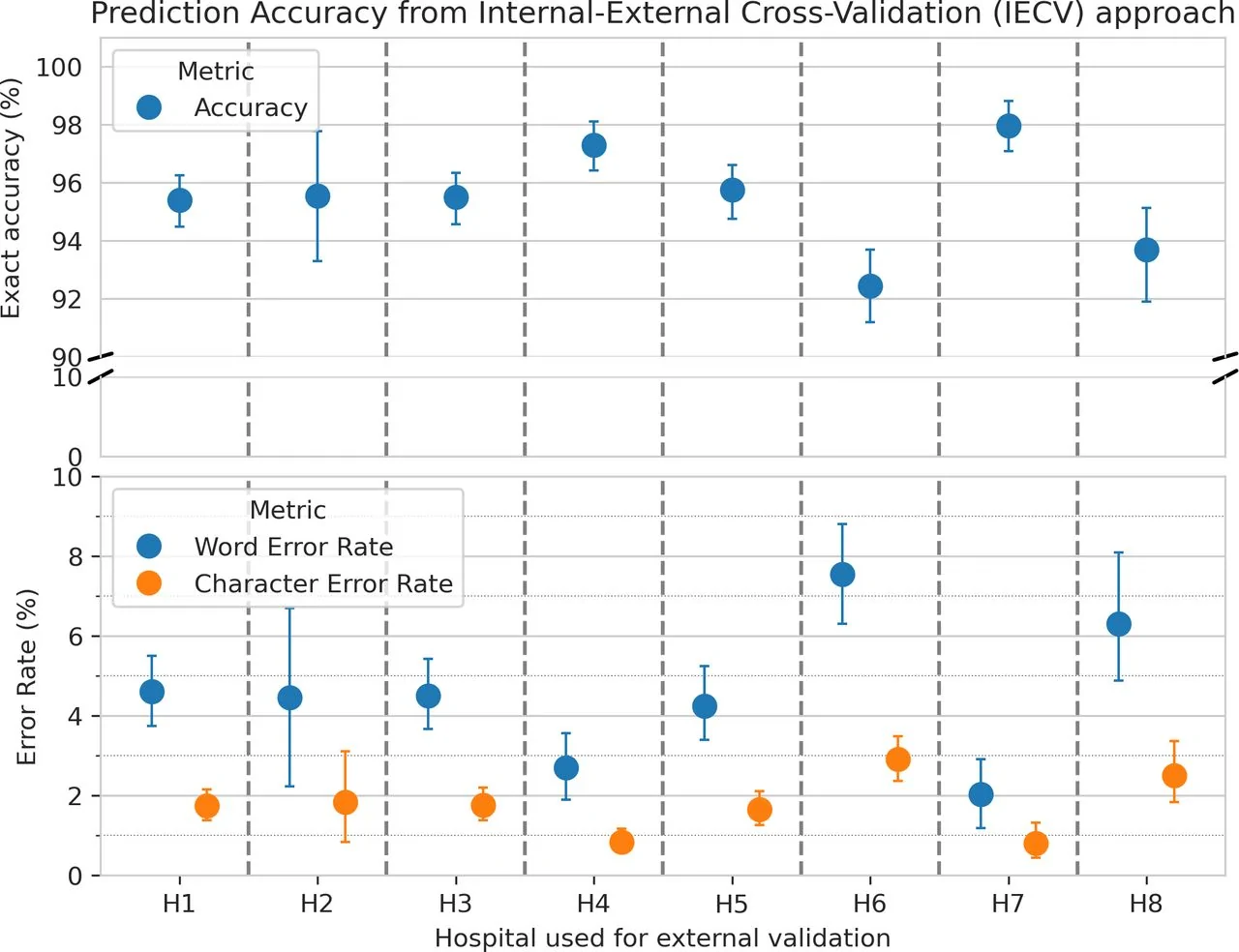
Model performance from IECV approach in predicting free-text fields. IECV, internal-external cross-validation.

The accuracy of the models in extracting free-text hand-written data was high across all metrics of interest, with the aggregate scores for exact accuracy, CER and WER across all hospitals being 95.66% (95% CI 94.24 to 96.74%), 1.66% (95% CI 1.21% to 2.26%) and 4.34% (95% CI 3.26% to 5.76%), respectively (table 1), despite the considerable variability between hospitals (figure 3).

### The co-designed clinical data pipeline

**Figure 4:**
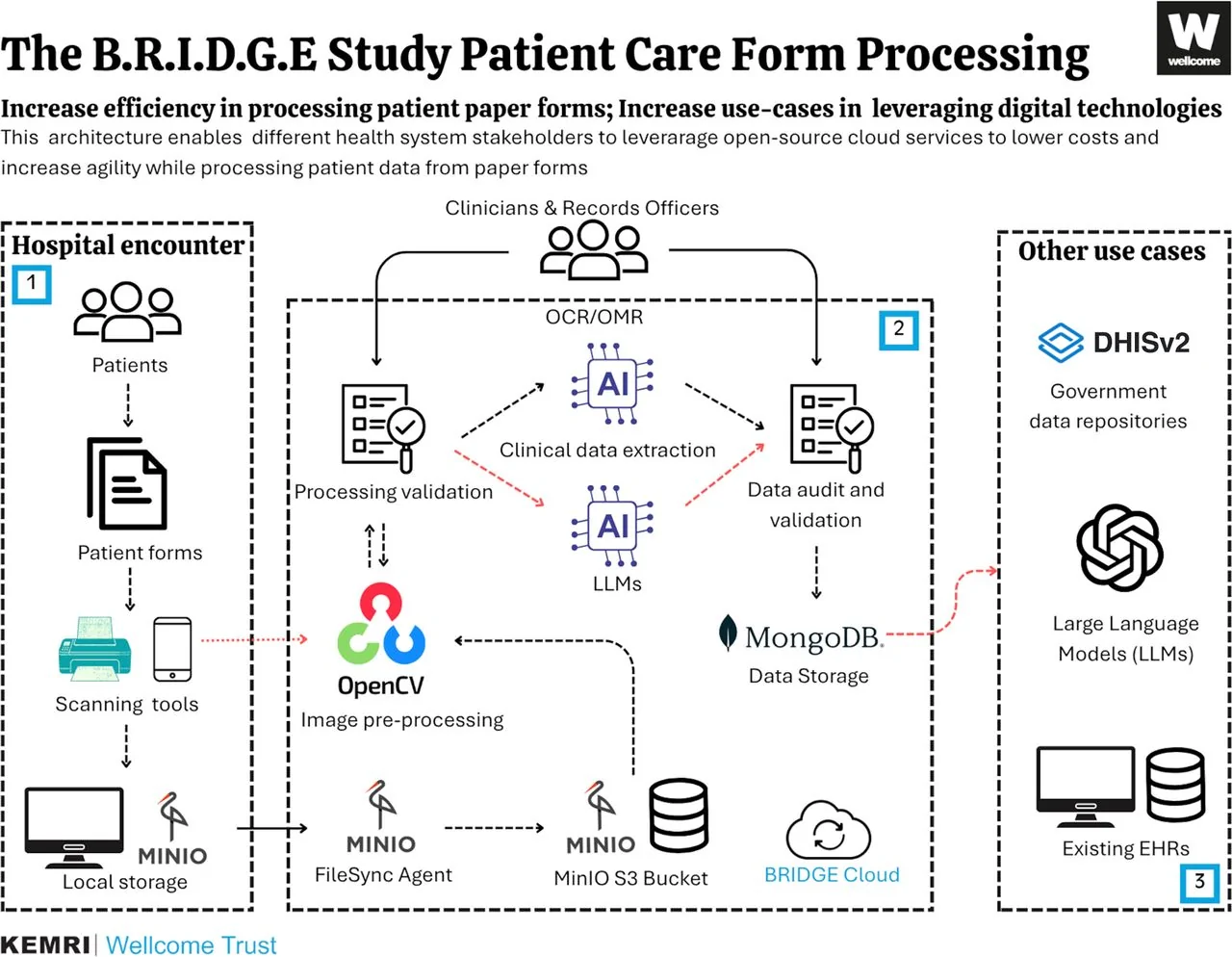
The co-designed interactive clinical data pipeline for processing paper forms using the novel paper-to-digital approach. The broken red-line illustrates extensible components of the pipeline for future development. AI, artificial intelligence; DHISv2, District Health Information System V.2; EHR, electronic health record; KEMRI, Kenya Medical Research Institute; OCR, Optical Character Recognition; OMR, Optical Mark Recognition.

From the human centred design (HCD) design process adapted from Boyd *et al*,^52^ we used a six-step process in two phases: design and piloting. Phase one began by understanding the data capture experience and exploring ideas on how to address the challenges identified. Next, we focused on practical solutions given the context and developed these ideas together with the HRIOs, nurses and doctors in the participating hospitals, leveraging the MoH-led stakeholder engagement efforts to institutionalise standardised newborn data capture and use (online supplemental table 1), resulting in a pipeline prototype (figure 4). In phase two, a study data manager iteratively piloted the developed pipeline prototype by uploading the 12 070 scans, adapting the pipeline based on any technical issues identified. While we describe the process sequentially, it was cyclical: stakeholders reflected on earlier suggestions on form design, data extraction and reporting and revised key steps with subsequent tests and feedback from piloting and as contextual issues became clearer.

The pipeline was designed to include interactive quality checks for the model-extracted data (unlike phase one’s human-extracted data) as the scanned image was being uploaded onto a digital repository, while also serving as a local digital archive of patient records. From a sample of 600 records, the time to auto-extract data using the developed models from a single page document with at least 40 variables using the pipeline is 4 min 30 s (SD: 2 min). From pilot exercises with 16 HRIOs during stakeholder engagement workshops (online supplemental table 1), it takes an average of 7 min (SD: 2 min) of human time to abstract the same data onto DHISv2 using the event tracker DHISv2 data-entry forms.

From DQA processes in the phase one data collection step of this study, when abstracting data from paper onto REDCap, HRIOs made data transcribing errors in 10 122/12 070 (83.86%) documents, with a median of two fields (IQR: 1–3) requiring correction. The models in the pipeline illustrated by figure 4 had fewer errors with only a third of the sampled documents (32.79%) requiring rectification with a median of two fields (IQR: 1–3) requiring correction, representing a ~60% reduction in documents with data requiring correction.

Based on feedback from clinicians, the pipeline was co-designed to be especially extensible for use with mobile devices like tablets (eg, using picture-to-scan functionality) to allow easier adoption and use with low-end smartphone devices. Furthermore, it was co-designed to leverage AI/ML models during the auto-extraction of data from scanned images while synchronously executing tasks like mortality risk score calculation or providing clinical decision support using large language models from use of the generated patient summaries (online supplemental table 4).

### Sustainability model and budget planning

The novel paper-to-digital innovation was designed to address current shortcomings of lack of quality patient-level clinical data from routine care due to the lack of widespread adoption of meaningful use of EHRs in typical hospital settings in SSA. The adoption of the hybrid paper-to-digital approach is thus expected to decline as meaningful adoption of EHRs gains momentum. The pipeline outlined in figure 4 was implemented with open-source tools that are freely available to the public to minimise operational costs due to licences. The software code, together with de-identified anonymised dataset, will be published separately on GitHub to make it easier for institutions to benefit from the contributions of other users/institutions while fostering knowledge sharing and community of practice around the innovation. Given it was piloted on low-cost scanners (~US$150), while designed to be extensible for use with smartphone devices that have cameras, the main cost of running this innovation is human resource costs of HRIOs.

### Sensitivity analyses

The models used in the highlighted pipeline approach for auto-extraction of data performed well even with as few records as 400 (online supplemental figure 4). Despite the considerable variance around the performance metrics estimates, all of the CIs were >95% indicating they were highly accurate and dependable in detecting the checkbox data entered on the forms. However, handwritten fields required considerably more documents to achieve high accuracy (online supplemental figure 5).

## Discussion

### Summary of findings

In this study, we report the design, implementation and evaluation of a novel hybrid paper-digital clinical data pipeline that uses AI to extract and report data from paper records, that is relatively easy to set up and deploy rapidly for use in routine clinical settings in resource-limited settings by clinical and records staff with minimal training and as a way to create complex registry data. The relative advantage of this approach illustrated in figure 4 is in the efficiency gains in ~36% reduction in the human time required to manually extract data from paper forms and report them onto routine reporting platforms like DHISv2 (or any other digital platform), and ~60% reduction in data capture errors.

### Comparison to other studies

While several studies have reported undertaking a similar approach to a thoughtful, context-sensitive integration of paper and digital systems, very few report AI model performance metrics from the data being auto-extracted from the paper medical records.^27 53 54^ Other studies constrain the paper form design to tabular format or custom proprietary designs to achieve arguably excellent performance of data auto-extraction using AI models, rendering the models largely untransferable to different paper form designs that are used in typical SSA hospital contexts.^28 54^ The paper forms in our study are publicly available and routinely used in typical hospital settings and are size A4 with at least 50 fields being collected per page, demonstrating considerably higher density of patient-level clinical data being extracted from a single image in comparison to other published studies.^27 53^ A significant shared similarity between our study and other studies is the use of HCD to ensure the adoption and sustainable use of the implemented digital tools while producing a shift in stakeholders’ mind-set from clinical data pipelines as data management tools to clinical data pipelines as quality of care instruments.^30 55^

### Implication of findings

The high model performance metrics indicate that the AI models can robustly and confidently identify checkbox selections and select key free-text in the machine-readable forms with minimal errors (table 1). From our key findings, the use of AI tools to automate clinical data flows might help produce the best use of clinicians’ and information officers’ time in a resource-constrained setting with especially high patient flows and limited human resources. Furthermore, such novel paper-to-digital clinical data pipelines make it easier not only to collect patient-level data to support resource allocation and quality improvement programmes at scale, but they also simplify the process of conducting pandemic or vaccine efficacy surveillance and/or carrying out research using hospital episodic data in remote rural areas, where the availability of infrastructural support (eg, internet, computers) coupled with trained clinical/record staff is limited.

With the patient-level data harnessed by the novel pipeline with relatively high accuracy, stakeholders can now leverage case-mix adjustment (ie, the statistical process to account for differences in the mix of patients across hospitals), in order to make fair comparisons of the relative performance of hospitals across geographies.

Fundamentally, our findings also imply that we are now able to use this approach iteratively during admission to auto-extract previously difficult-to-get longitudinal patient-level treatment data from treatment sheets in patient files that are useful for modelling and reporting patterns and trends in antimicrobial stewardship and resistance.^56^

### Strengths and limitations of the study

A key strength of this study is that evaluating the novel paper-to-digital approach was done with >7000 hospital admission encounters across eight hospitals, involving both structured (eg, the REDCap dataset) and unstructured (ie, the scanned images) clinical datasets. Such data from routinely-used paper tools in typical clinical settings from geographically dispersed hospitals for AI modelling are quite uncommon in SSA.^57^ Additionally, we implemented IECV at the hospital level and reported diverse model performance metrics which allowed us to characterise the AI models’ generalisability to other neonatal units and better address heterogeneity in model performance across populations.^46^

A key challenge of this study is that while it demonstrates design thinking considerations in several ways to maximise information acquisition, information use—whether for goal setting, resource allocation or performance tracking, etc—still lags behind^58^; furthermore, with the use of AI models for data extraction, the acceptability of the models’ margin of error in extracted data accuracy (as demonstrated by the CIs in figures 3 and 4) by the intended recipients of the data is yet to be systematically evaluated. Methodologically, AI model performance is highly dependent on the quality of paper used for clinical documents, with carbon copy paper types demonstrating very low readability (even for humans) hindering the use of hybrid paper-to-digital approaches. However, the novel pipeline has been designed to allow for future work that can explore use of cameras in smartphone devices to collate clinical information during an ongoing hospital encounter contributing to a ‘living record’ with clinicians having access to digitised data before patient discharge.

## Conclusion

This study shows that a co-designed paper-to-digital pipeline that uses simple AI models can extract patient-level clinical data from routine paper records with over 94% accuracy while halving the time required for manual abstraction. This approach offers a practical, scalable solution for generating individual-level patient-level data or complex register-type data needed for case-mix adjustment, quality benchmarking and disease surveillance in settings such as SSA where comprehensive EHR implementation remains out of reach, especially in inpatient care settings. Determining acceptable accuracy thresholds among end-users and identifying sustainable implementation models are priorities for future research.

## Data Availability

Data are available upon reasonable request. The datasets generated and/or analysed during the current study are not publicly available due to the primary data being owned by the hospitals and their counties with the Ministry of Health. The research staff do have permission to share the data without further written approval from both the KEMRI-Wellcome Trust Data Governance Committee and the Facility, County or Ministry of Health as appropriate to the data request. Requests for access to primary data from qualitative research by people other than the investigators will be submitted to the KEMRI-Wellcome Trust Research Programme data governance committee as a first step through dgc@kemri-wellcome.org, who will advise on the need for additional ethical review by the KEMRI Research Ethics Committee.
