# Supplementary material for: Using a novel hybrid AI-based paper-to-digital pipeline for extracting patient-level clinical data from routine hospital records in Kenya: a retrospective multicentre study
Source: BMJ Digit Health Ai. 2026 May 22;2(1):e000092. doi: 10.1136/bmjdh-2026-000092 (PMC13528387; doi:10.1136/bmjdh-2026-000092)
Supplement: Supplementary file 1 [file bmjdh-2-1-bmjdh-2026-000092-s001.pdf]

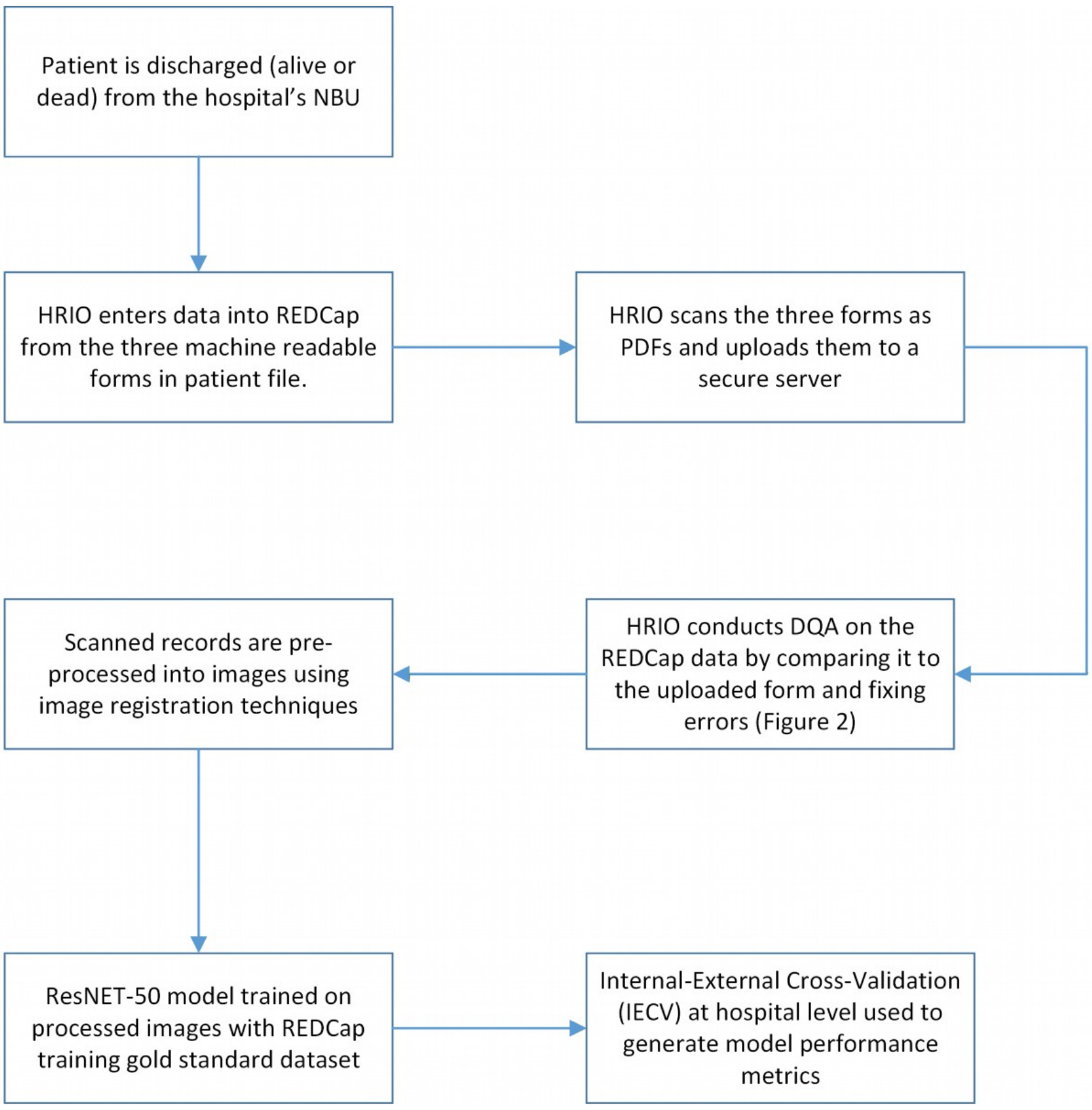

Supplementary Figure 1: Summary of the data capture, management and modelling process for the BRIDGE study

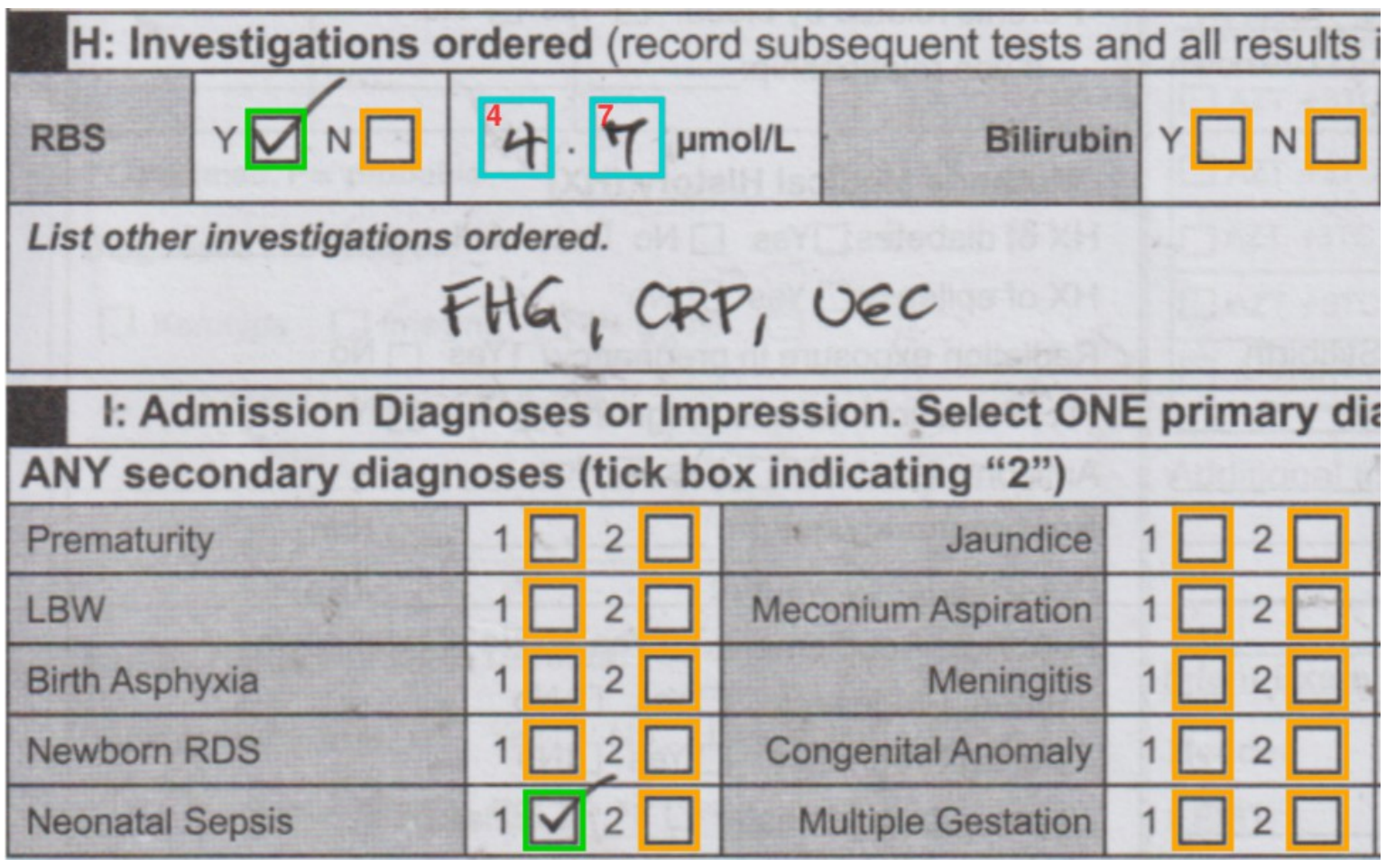

Supplementary Figure 2: Data quality assurance checks before model building for the BRIDGE study

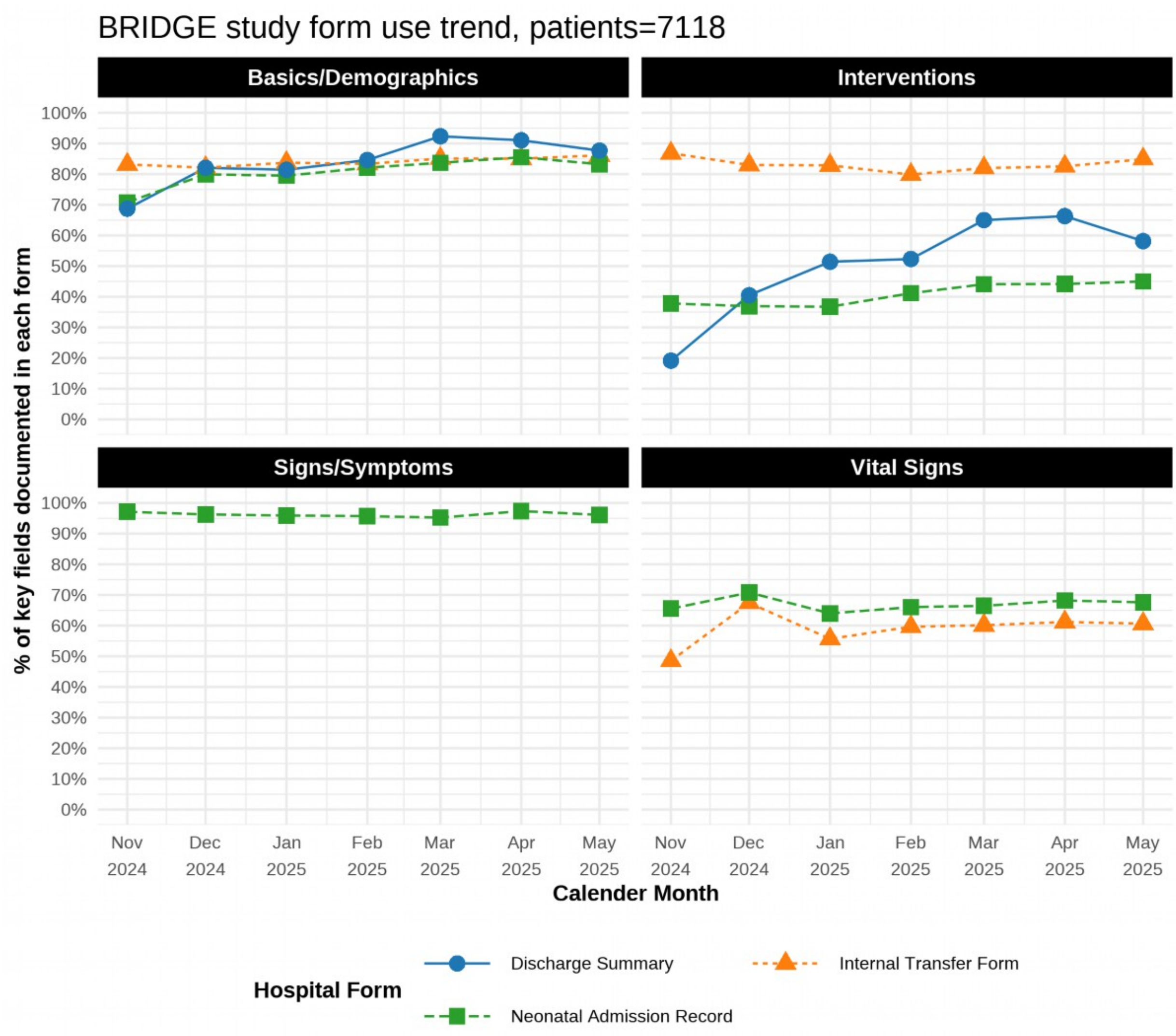

Basics/Demographics key variables: Sex, age (days), Apgar@5m, birth weight, admission weight, discharge weight*, born outside, delivery type, admission date, date of birth, gestation (in weeks)

Vitals key variables: Pulse oximetry, pulse rate, blood pressure, respiratory rate, temperature, random blood sugar

Signs/Symptoms key variables: difficulty breathing, difficulty feeding, floppy, grunting, convulsions, crackles, central cyanosis, lower chest indrawing

Interventions key variables: CPAP, phototherapy, oxygen, antibiotics, IV fluids, KMC, BVM resuscitation, caffeine citrate, nutrition/feeds

Supplementary Figure 3: Summary of the key vital demographic, symptoms and vital signs and interventions captured by the routine hospital forms in the BRIDGE study

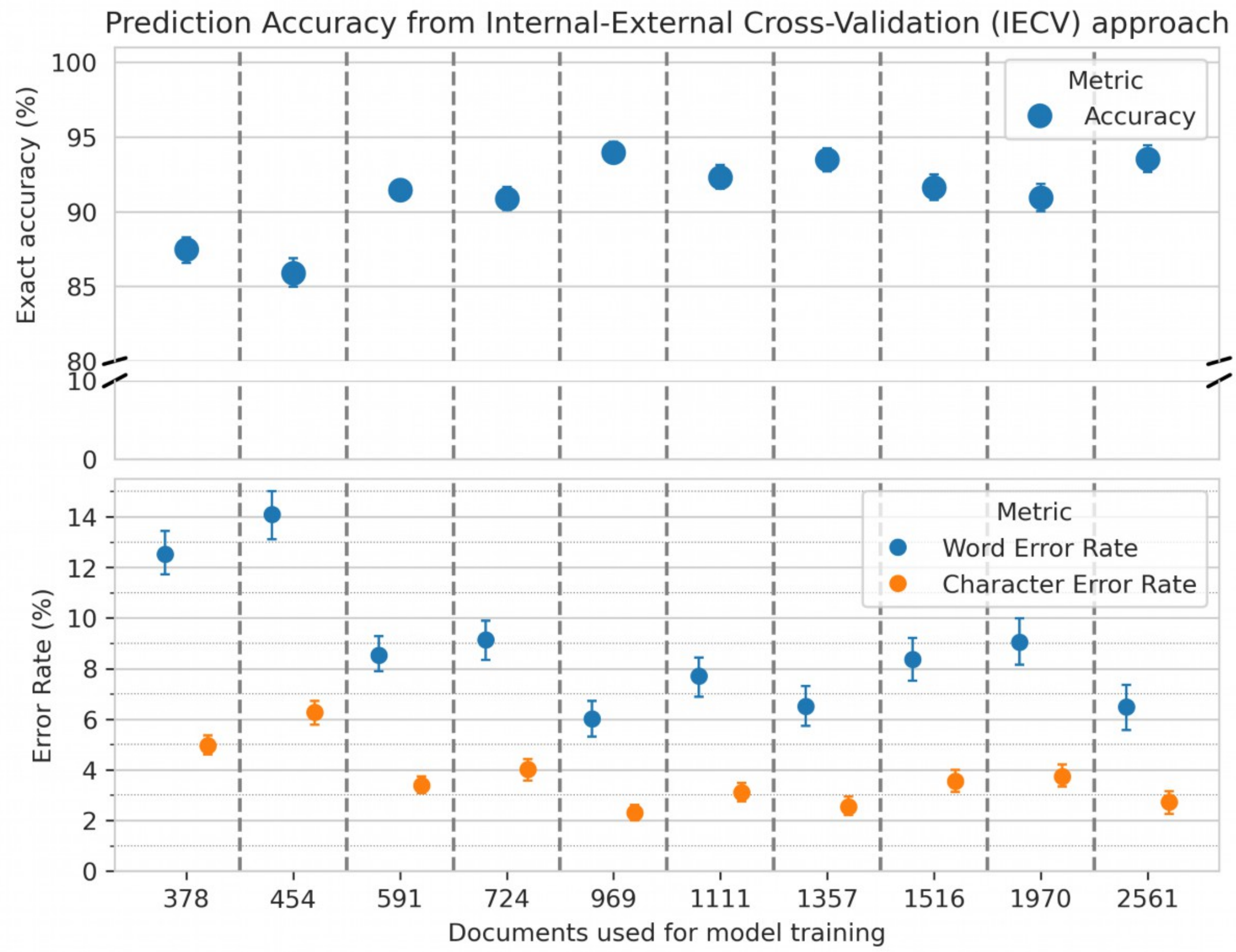

Supplementary Figure 4: Model performance heterogeneity for checkbox fields based on number of documents used

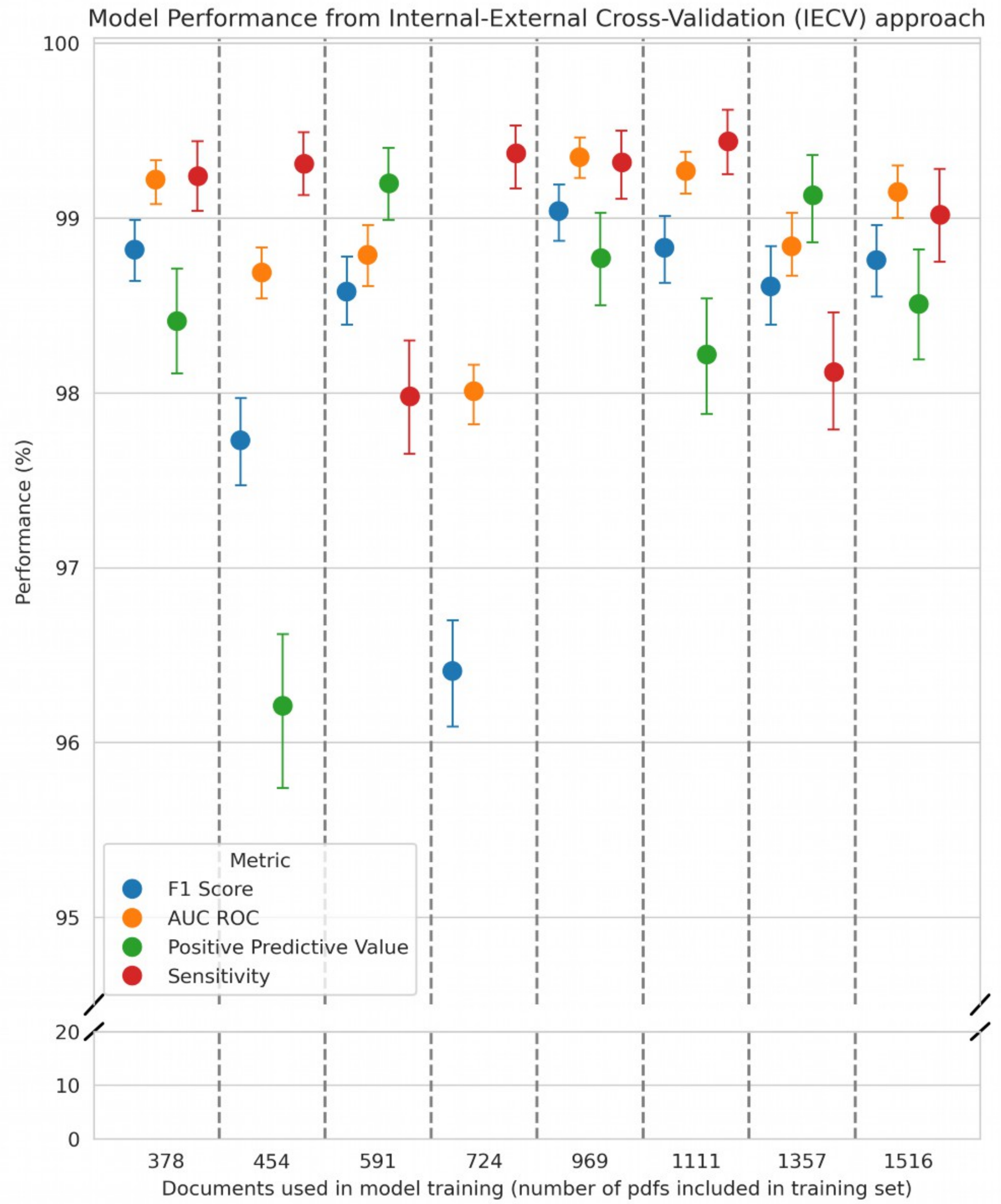

Supplementary Figure 5: Model performance heterogeneity for free-text fields based on number of documents used
