# Supplementary material for: Using a novel hybrid AI-based paper-to-digital pipeline for extracting patient-level clinical data from routine hospital records in Kenya: a retrospective multicentre study
Source: BMJ Digit Health Ai. 2026 May 22;2(1):e000092. doi: 10.1136/bmjdh-2026-000092 (PMC13528387; doi:10.1136/bmjdh-2026-000092)
Supplement: Supplementary file 2 [file bmjdh-2-1-bmjdh-2026-000092-s002.pdf]

Supplementary Tables

Supplementary Table 1: Roadmap to the standardisation, scale-up, and digitisation of patient-level data collection in Kenya	
**Dates**	**Activities/ Milestones**
**26th to 28th February 2024**	Workshop on digitisation of national quality of care standards and neonatal core indicator dataset development
**27th May to 31st May 2024**	Workshop to finalise draft newborn and child Health paper tools for piloting
**01st July to 30th September 2024**	Piloting of newborn and child health paper tools in health facilities
**02nd to 04th October 2024**	Newborn and child health paper tools validation meeting from pilot findings
**14th November 2024**	Official national launch of the newborn and child health inpatient booklets
**09th to 13th June 2025**	Patient-level neonatal DHISv2 data integration workshop: Co-design of the digital data collection tools
**22nd to 26th September 2025**	Patient-level neonatal DHISv2 data integration workshop: Piloting of the digital patient-level data reporting tools on DHISv2
**November 2025**	DHISv2 neonatal event tracker piloting in one County Referral Hospital
**16th to 20th February 2026**	Configuring the newborn module into the Kenyan national EHRs system (i.e. Taifa Care) and finalise the newborn dashboard integration to strengthen data use for evidence-based decision making at all levels
**Note:** The list of stakeholders involved the Ministry of Health (MoH), UNICEF country office, WHO country office, Save the Children, KEMRI-Wellcome Trust Research Programme, The NEST360 programme, the Kenya Paediatric Association (KPA), The Clinton Health Access Initiative (CHAI), The Kenya Paediatric Research Consortium (KEPRECON) and paediatricians and HRIOs from select referral-level hospitals	

Supplementary Table 2: Scanned image pre-processing steps	
**Step**	**Description**
1) Image preprocessing	Optical fiducial mark detection, extract the main document area
2) Image registration	Uploaded scan aligned to the paper template design it was based on
3) Regions of interest (ROI) extraction	Snippets of the areas of interest for data extraction are isolated and linked to REDCap data entered in those sections
4) ROI preparation for modelling	The ROIs are re-shaped to the same dimensions for modelling tasks

<table>
<tr><th colspan="2">Supplementary Table 3: Model fitting procedures</th></tr>
<tr><td>Images preprocessing</td><td>OpenCV version 4.7.0</td></tr>
<tr><td>Initial OpenCV Image Preprocessing Objective</td><td>This was done on the images of the medical records to improve image quality prior to downstream extraction and analysis.</td></tr>
<tr><td>OpenCV preprocess steps</td><td>1. Convert to grayscale
2. Denoise
3. Contrast enhancement
4. Adaptive thresholding
5. Morphological cleanup
6. Alignment correction</td></tr>
<tr><td>Model</td><td>ResNet50 from tf.keras.applications.resnet50.ResNet50</td></tr>
<tr><td>Model library</td><td>TensorFlow 2.15.0 with Keras backend version 2.15.0</td></tr>
<tr><td>Model fine-tuning specification</td><td>Model exposes 177 layers, all of which can be retrained/fine-tuned (not just the 50 weighted layers). We unfroze the model after the 25th layer which, technically, made 92 layers with trainable weights available for fine-tuning after omitting Activation, Add, ZeroPadding2D and MaxPooling2D layers that have no trainable weights.</td></tr>
<tr><td rowspan="2">Fitted model structures</td><td>
<table>
<tr><th colspan="3">OMR Model: "omr_transfer_resnet50_finetune"</th></tr>
<tr><th>Layer (type)</th><th>Output shape</th><th>Param #</th></tr>
<tr><td>preprocess (Lambda)</td><td>(None, 48, 48, 3)</td><td>0</td></tr>
<tr><td>resnet50 (Functional)</td><td>(None, 2, 2, 2048)</td><td>23587712</td></tr>
<tr><td>Average_2D (GlobalAveragePooling 2D)</td><td>(None, 2048)</td><td>0</td></tr>
<tr><td>dropout (Dropout) (50%)</td><td>(None, 2048)</td><td>0</td></tr>
<tr><td>prediction (Dense)</td><td>(None, 1)</td><td>2049</td></tr>
<tr><td colspan="3">Total params: 23589761 (89.99 MB)
Trainable params: 23397761 (89.26 MB)
Non-trainable params: 192000 (750.00 KB)</td></tr>
</table>
</td></tr>
<tr><td>
<table>
<tr><th colspan="3">OCR Model: "ocr_transfer_resnet50_finetune"</th></tr>
<tr><th>Layer (type)</th><th>Output shape</th><th>Param #</th></tr>
<tr><td>resize_to_48X48 (Resizing)</td><td>(None, 48, 48, 3)</td><td>0</td></tr>
<tr><td>preprocess (Lambda)</td><td>(None, 48, 48, 3)</td><td>0</td></tr>
<tr><td>resnet50 (Functional)</td><td>(None, 2, 2, 2048)</td><td>23587712</td></tr>
<tr><td>Average_2D (GlobalAveragePooling 2D)</td><td>(None, 2048)</td><td>0</td></tr>
<tr><td>dropout (Dropout) (50%)</td><td>(None, 2048)</td><td>0</td></tr>
<tr><td>flatten (Flatten)</td><td>(None, 2048)</td><td>0</td></tr>
<tr><td>hold_info (Dense)</td><td>(None, 128)</td><td>262272</td></tr>
<tr><td>prediction (Dense)</td><td>(None, 14)</td><td>1806</td></tr>
<tr><td colspan="3">Total params: 23851790 (90.99 MB)
Trainable params: 23659790 (90.25 MB)
Non-trainable params: 192000 (750.00 KB)</td></tr>
</table>
</td></tr>
<tr><td>Hyper-parameter tunning</td><td>Using the Internal-External Cross Validation (IECV) approach:
Step 1: One hospital at a time held out as the external validation dataset
Step 2: Model built the on the dataset of the remaining hospitals
• Use 30% of the images from the training dataset for internal validation to avoid overfitting and allow early stopping after each epoch
Step 3: Model performance evaluated on the data from the hospital held out.</td></tr>
</table>

**Supplementary Table 4: Generatable patient summaries collated for key variables in the admission record using the clinical data pipeline**	
Short summary	Long summary (indicative of missingness)
Demographics (all variables = 9):<br>• sex: female<br>• blood group: unknown<br>• rhesus: unknown<br>• encounter: 8_760001046<br><br>Presenting (all variables = 10):<br>• apnoea: has no apnoea<br>• convulsions: has no convulsions<br>• diarrhoea: has no diarrhoea<br>• difficulty breathing: has difficulty breathing<br>• difficulty feeding: has no difficulty feeding<br>• fever: has no fever<br>• vomiting: has no vomiting<br>• floppy: is not floppy<br><br>Past medical history (all variables = 9):<br>• apgar 1m: 5<br>• apgar 5m: 6<br>• apgar 10m: 7<br>• delivery type: svd<br>• gestation in weeks: 32<br><br>Vitals (all variables = 7):<br>• temperature: 36.0<br>• respiratory rate: 22<br>• pulse oximetry: 87<br>• pulse rate: 136<br><br>Anthropometrics (all variables = 3):<br>• admission weight in grams: 1038<br><br>Investigations (all variables = 20):<br>• capillary refill in seconds: 3<br>• chest indrawing: yes<br>• cry: weak<br>• bulging fontanelle: has no bulging fontanelle<br>• central cyanosis: has no central cyanosis<br>• crackles: has no crackles<br>• good air entry: has good air entry<br>• grunting: has no grunting<br>• murmur: has no murmur<br>• intercostal retraction: mild<br>• xiphoid retraction: mild<br>• distended: is not distended<br>• irritable: is not irritable<br>• pallor: none	Demographics (all variables = 9):<br>• baby age in days: missing<br>• sex: female<br>• blood group: unknown<br>• rhesus: unknown<br>• encounter: 8_760001046<br><br>Presenting (all variables = 10):<br>• apnoea: has no apnoea<br>• convulsions: has no convulsions<br>• diarrhoea: has no diarrhoea<br>• difficulty breathing: has difficulty breathing<br>• difficulty feeding: has no difficulty feeding<br>• fever: has no fever<br>• vomiting: has no vomiting<br>• floppy: is not floppy<br><br>Past medical history (all variables = 9):<br>• born before arrival: missing<br>• birth weight: missing<br>• apgar 1m: 5<br>• apgar 5m: 6<br>• apgar 10m: 7<br>• delivery type: svd<br>• gestation in weeks: 32<br><br>Vitals (all variables = 7):<br>• temperature: 36.0<br>• respiratory rate: 22<br>• pulse oximetry: 87<br>• pulse rate: 136<br>• systolic blood pressure: missing<br>• diastolic blood pressure: missing<br><br>Anthropometrics (all variables = 3):<br>• length: missing<br>• head circumference: missing<br>• admission weight in grams: 1038<br><br>Investigations (all variables = 20):<br>• capillary refill in seconds: 3<br>• chest indrawing: yes<br>• cry: weak<br>• birth defects: missing<br>• bulging fontanelle: has no bulging fontanelle<br>• central cyanosis: has no central cyanosis<br>• crackles: has no crackles

• jaundice: none<br>• umbilicus: clean<br>• skin: normal<br><br>Diagnoses (all variables = 2):<br>• primary admission diagnosis: prematurity, rds<br>• secondary admission diagnosis: lbw<br><br>Interventions (all variables = 12):<br>• prescribed antibiotics: yes<br>• prescribed cpap: yes<br>• prescribed iv fluids: yes	• good air entry: has good air entry<br>• grunting: has no grunting<br>• murmur: has no murmur<br>• intercostal retraction: mild<br>• xiphoid retraction: mild<br>• distended: is not distended<br>• irritable: is not irritable<br>• pallor: none<br>• jaundice: none<br>• umbilicus: clean<br>• skin: normal<br><br>Diagnoses (all variables = 2):<br>• primary admission diagnosis: prematurity, rds<br>• secondary admission diagnosis: lbw<br><br>Interventions (all variables = 12):<br>• prescribed antibiotics: yes<br>• prescribed caffeine citrate: missing<br>• prescribed cpap: yes<br>• prescribed feeds: missing<br>• prescribed incubator: missing<br>• prescribed iv fluids: yes<br>• prescribed kmc: missing<br>• prescribed opv: missing<br>• prescribed oxygen: missing<br>• prescribed phototherapy: missing<br>• prescribed surfactant: missing<br>• prescribed transfusion: missing
